# Environmentally relevant aged nanoplastics amplify oxidative stress–associated inhalation toxicity and delay lung clearance

**DOI:** 10.1016/j.redox.2026.104281

**Published:** 2026-06-27

**Authors:** Soyeon Jeon, Jun Hui Jeon, Gyuri Kim, Sung Ik Yang, Wan-Seob Cho

**Affiliations:** a Lab of Toxicology, Department of Health Sciences, The Graduate School of Dong-A University, 37, Nakdong-daero 550 beon-gil, Saha-gu Busan, 49315, Republic of Korea; b Department of Applied Chemistry, Kyung Hee University, Yongin-si, 17104, Republic of Korea

**Keywords:** Inhalation toxicity, Secondary microplastics, Oxidative stress, Reactive oxygen species, Lung burden

## Abstract

Airborne micro- and nanoplastics are emerging environmental contaminants of increasing concern, yet their inhalation toxicity and pulmonary fate remain insufficiently understood. In this study, we investigated how particle morphology and surface oxidation influence pulmonary inflammation and clearance using environmentally relevant, size-controlled polystyrene (PS) nanoplastics. Spherical and fragmented PS were generated via bottom-up and top-down approaches, respectively, and subjected to ultraviolet irradiation to simulate environmental aging. Fragmented and ultraviolet (UV)-aged nanoplastics exhibited enhanced surface oxidation and a higher intrinsic oxidative potential than pristine spherical particles. Following a single pulmonary exposure in mice via pharyngeal aspiration at doses of 25–100 μg per mouse, these environmentally transformed nanoplastics induced more severe acute pulmonary inflammation. In particular, UV-aged fragmented PS (100 μg per mouse) increased neutrophil counts by 3.9-fold compared with pristine spherical PS, along with increased pro-inflammatory cytokine production, which was closely associated with particle-derived oxidative reactivity. Although acute inflammatory responses were largely reversible, time-course analysis of lung burden after a single non-overload exposure (25 μg per mouse) revealed delayed pulmonary clearance of fragmented nanoplastics relative to spherical particles, with estimated clearance half-lives of 13.5 days for pristine spherical PS and 27.4 days for UV-aged fragmented PS. Overall, this study demonstrates that nanoplastics most relevant to real-world environmental exposure may pose a greater risk of respiratory health effects, along with prolonged lung residence.

## Highlights

• Environmental transformation alters the pulmonary toxicity of airborne nanoplastics.
• Fragmentation and UV aging elevate particle-derived reactive oxygen species.
• Oxidative stress is closely associated with acute lung inflammatory responses.
• Plastic morphology critically affects lung clearance under non-overload conditions.
• Pristine particles might underestimate the pulmonary toxicity of nanoplastics.

## Graphical abstract

**Figure:**
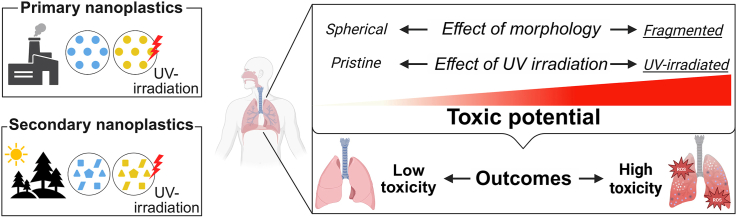

## 1 Introduction

Nanoplastics have recently emerged as a new class of airborne pollutants, raising growing concerns about their potential respiratory toxicity [1,2]. Nanoplastics are commonly classified into primary nanoplastics, intentionally manufactured, and secondary nanoplastics, generated through environmental weathering of larger plastic debris [3]. However, most inhalation toxicity studies to date have focused on primary particles because of their well-defined physicochemical properties and the relative ease of particle labeling and surface modification for tracking and distribution analyses [1,4]. Although such studies provide valuable mechanistic insight, they leave a critical knowledge gap regarding inhalation-relevant exposure scenarios in which environmentally derived secondary nanoplastics predominate [5,6]. Therefore, there is an urgent need to investigate the inhalation toxicity and biodistribution of secondary nanoplastics, which are more environmentally relevant yet poorly understood.

Environmental monitoring consistently indicates that polystyrene (PS) constitutes a relatively small proportion of total environmental microplastics compared with polyethylene (PE) and polypropylene (PP) [7]. Nevertheless, expanded polystyrene (EPS), commonly known as Styrofoam, may dominate in specific contexts, particularly in regions with high packaging waste and coastal discharge zones [8]. EPS is lightweight, highly porous, and widely used for packaging and insulation; however, its environmental persistence, poor recyclability, and potential release of toxic monomers raise significant concerns [4]. Despite its widespread environmental occurrence, the toxicological implications of EPS-derived airborne particles remain largely unexplored. Given that EPS fragments constitute a major source of floating and airborne microplastics, there is an urgent need to assess their pulmonary toxicity under realistic exposure conditions.

Nanoplastics are insoluble particles that can induce persistent pulmonary toxicity upon respiratory exposure by continuously stimulating macrophages and epithelial cells [9,10]. The underlying mechanisms of micro- and nanoplastics-induced toxicity involve cellular oxidative stress and the release of proinflammatory cytokines, including interleukin (IL)-1β and tumor necrosis factor-α (TNF-α) [10,11]. Fragmented or needle-like micro- and nanoplastics derived from environmental processes may elicit comparable inflammatory pathways following inhalation exposure.

Similar to poorly soluble nanoparticles, micro- and nanoplastics can persist in the lung due to inefficient lysosomal degradation within macrophages, leading to prolonged particle–cell interactions [9,12,13]. Therefore, quantitative analysis of particles retained in pulmonary tissues is critical for accurate assessment of their toxicity. However, quantitative assessment of pulmonary retention and clearance has largely depended on artificial labeling approaches, and the applicability of these methods to environmentally aged or secondary microplastics remains uncertain [14,15]. Therefore, this study investigated lung inflammation and pulmonary clearance kinetics induced by primary PS and secondary EPS, with or without ultraviolet (UV) irradiation, to better assess realistic respiratory hazards associated with nanoplastics.

## 2 Materials and methods

### 2.1 Synthesis of primary and secondary PS nanoplastics

Pristine spherical PS nanoplastics (PSPS) were synthesized via a bottom-up strategy using nano-emulsion polymerization. Briefly, the stabilizer, 4-tert-butylpyrocatechol, present in styrene was first removed using aluminum oxide, after which sodium dodecyl sulfate was added at a final concentration of 20% as a surfactant. Potassium persulfate was then added as an initiator, and the mixture was stirred thoroughly. The reaction temperature was subsequently raised to 100 °C, followed by the addition of styrene and ethanol, the latter acting as a cosurfactant. Polymerization was carried out for 18 h under these conditions. After completion, the reaction mixture was filtered through a cell strainer to remove residual impurities, then centrifuged at 7441 × *g* to discard the supernatant. The resulting pellet was washed with deionized water and dried under vacuum at room temperature for 24 h to obtain purified primary PS nanoplastics. In contrast, pristine fragmented PS nanoplastics (PFPS) were prepared using a top-down approach, in which disposable Styrofoam plates were ball-milled as previously described in our study [10].

### 2.2 UV-induced weathering of nanoplastics

To simulate an oxidation-driven aspect of environmental nanoplastic weathering, an accelerated UV aging protocol was employed following the American Society for Testing and Materials (ASTM) G154-12, which describes standardized operating procedures for fluorescent UV lamp exposure in controlled laboratory conditions [16]. This approach was not intended to fully replicate the complexity of natural environmental aging, which may involve multiple concurrent processes such as wet-dry cycling, microbial transformation, and mechanical abrasion, but rather to provide a reproducible and well-defined model of UV-induced surface oxidation. Briefly, PSPS and PFPS were dispersed in deionized water and gently agitated throughout the exposure period to ensure uniform irradiation and oxidation across particle surfaces. The suspensions were then placed in a custom-built UV exposure chamber fabricated in accordance with ASTM specifications and subjected to UV-B irradiation at 306 nm (Shinko Denki, Japan). The UV-induced oxidation was performed at ambient temperature for 14 days. After irradiation, the nanoplastics were collected, washed, and prepared for subsequent analyses. The UV-treated particles were designated as UV-irradiated spherical PS nanoplastics (USPS) and UV-irradiated fragmented PS nanoplastics (UFPS).

### 2.3 Analysis of physicochemical properties of test nanoplastics

#### 2.3.1 Measurement of size, morphology, surface charge, and chemical composition

The primary size and morphology of the test nanoplastics were measured using high-resolution field-emission scanning electron microscopy (FE-SEM; LEO SUPRA 55, Carl Zeiss, Germany) and high-resolution field-emission transmission electron microscopy (FE-TEM; JEM-2100F, JEOL, Japan). The zeta potential and hydrodynamic diameter (Dh) were determined using a Zetasizer Nano ZS-90 (Malvern Panalytical, Malvern, UK) in phosphate-buffered saline (PBS) after the dispersion process. The specific surface area and total pore volume of the nanoplastics were analyzed using the Brunauer–Emmett–Teller (BET) method with a BELSORP-max instrument (Microtrac, York, PA, USA). The chemical composition of the nanoplastics was evaluated using confocal Raman spectroscopy (UniRAM-3200 Micro Raman system, Uni-nano Tech; Yong-in, Korea) with laser wavelengths of 532 nm and 633 nm. In addition, the C1s spectrum was analyzed using X-ray Photoelectron Spectroscopy (XPS).

#### 2.3.2 Measurement of the oxidative potential of nanoplastics

To evaluate whether the intrinsic oxidative reactivity of nanoplastics contributes to their pulmonary toxicity, we assessed the particle-associated oxidative reactivity of the test nanoplastics using a 2′,7′-Dichlorodihydrofluorescein diacetate (DCFH-DA)-based assay as previously described [10]. Briefly, 1 part of DCFH-DA was reacted with 40 parts of 0.01 M NaOH for 30 min at 25 °C. The suspension was mixed with 200 parts of 25 mM phosphate-buffered saline (PBS) and then stored at 4 °C prior to use. The test nanoplastics suspension was prepared at 2× the final concentration, as required by the reaction, to achieve a 1:1 ratio with the DCFH-DA suspension. Then, horseradish peroxidase (HRP) was added to the mixture at a final concentration of 2.2 units/mL, followed by reaction with the nanoplastics suspension at a 1:1 ratio. The solution was incubated in a water bath at 37 °C for 15 min and then centrifuged at 21,000 × *g* for 30 min to remove the nanoplastics. The supernatants were measured for fluorescence at 485/520 nm using a microplate reader (BioTek, Winooski, VT, USA). Reactive oxygen species (ROS) levels were expressed as hydrogen peroxide (H_2_O_2_) equivalents to determine the intrinsic ROS of the nanoplastics. A standard calibration curve was generated using H_2_O_2_ standards at concentrations of 0, 1.25, 2.5, 5, and 10 μM, and ROS levels were quantified by linear regression fitting of the standard curve.

### 2.4 Dispersion of test nanoplastics for *in vivo* studies

Because the hydrophobic nature and low density of the test particles led to poor dispersion in distilled water (DW) [17], heat-inactivated BALB/c serum obtained from four mice was used as the dispersing medium, as previously described [10]. Briefly, all test materials were prepared in individual tubes at each target concentration and first mixed with heat-inactivated BALB/c serum at 3% (*v/v*) of the final working volume, followed by sonication for 10 min using a bath sonicator (Saehan Sonic, Gyeonggi-do, Korea). After sonication, DW was added to constitute the remaining 97% of the final working volume, yielding a dispersion containing 3% mouse serum. The suspension was sonicated for 10 min using a bath sonicator (Saehan Sonic). To remove residual serum components, the samples were washed through three cycles of centrifugation (21,000 × *g*, 30 min) and resuspension of the pellets in 1 mL of DW, followed by sonication for 10 min. Finally, the pellets were resuspended in PBS at the designated treatment concentrations.

### 2.5 Pharyngeal aspiration of test nanoplastics to BALB/c mice

Six-week-old female BALB/c mice were purchased from Hana Biotech (Gyeonggi-do, Korea). After a 7-day acclimatization period, 7-week-old female mice were used for pharyngeal aspiration to maintain consistency with our established pulmonary exposure model [18]. The animal study was conducted in accordance with the guidelines of the Institutional Animal Care and Use Committee at Dong-A University (approval number: DIACUC-25-16). The mice were acclimated under conditions (humidity: 40-60%, temperature: 22-24 °C, 12 h light/dark cycle). After acclimatization, the mice were anesthetized with isoflurane (Hana Pharm Co., Ltd., Seoul, Korea) using a rodent anesthesia system (VetEquip, Pleasanton, CA, USA). While under anesthesia, the tongue was gently but firmly extended to expose the oropharynx, and 50 μL of the prepared suspension was placed at the base of the tongue, with the mouse positioned in a near-vertical orientation on a support board. The animals were then allowed to recover spontaneous breathing, during which the suspension was aspirated into the respiratory tract.

The treatment doses were selected as 25, 50, and 100 μg/mouse. The multiple-path particle dosimetry (MPPD) (v3.04) modeling suggested that an alveolar deposition of 100 μg of 500 nm particles in mice was estimated to be roughly comparable to the cumulative dose expected from repeated inhalation exposure to particulate matter with an aerodynamic diameter ≤2.5 μm (PM_2.5_) at 1 mg/m^3^, 8 h/day, for approximately 17 days, a concentration reported in Seoul [19]. The MPPD model was used in this study not to predict exact equivalence to real-world environmental exposure, but to provide an inhalation-relevant dosimetric context for the aspiration dose; however, interpretation should be cautious for low-density and heterogeneous nanoplastics, whose inhalation behavior may not be fully captured by simplified model assumptions. The reversibility of lung inflammation was assessed exclusively at the highest dose (100 μg/mouse), while lung clearance kinetics were evaluated at the low dose (25 μg/mouse) to minimize the influence of inflammation on particle clearance. PBS served as the vehicle control (VEH). Four mice were assigned to each group in both the toxicity assessment and lung clearance study.

### 2.6 Evaluation of lung inflammation at days 1 and 28 post-exposure

On days 1 and 28 post-exposure, the mice were euthanized by exsanguination via incision of the inferior vena cava under deep anesthesia using a rodent anesthesia system (VetEquip), and pulmonary inflammation was evaluated by bronchoalveolar lavage fluid (BALF) analysis. For BALF collection, the pleural cavity was opened, and a 22-gauge plastic catheter was inserted into the trachea and secured with an elastic thread. Then, the catheter was connected to a 1 mL syringe containing 700 μL of cold PBS, which was gently instilled into the lungs and subsequently collected by gentle massage. This lavage procedure was repeated four times, after which the BALF samples were centrifuged at 375 × *g* for 5 min. The supernatant from the first BALF fraction was collected separately for biological analysis, whereas the supernatants from the remaining three BALF fractions were discarded. Cell pellets from all BALF fractions were then pooled and resuspended in 1 mL of complete Roswell Park Memorial Institute (RPMI)-1640 medium (WELGENE, Gyeongsangbuk-do, Korea) supplemented with 10% fetal bovine serum (FBS) (Corning, Corning, NY, USA). Total cell counts were determined using a NucleoCounter (Chemometec, Allerød, Denmark). For differential cell counting, 4 × 10^4^ cells per slide were deposited onto glass slides using a cytospin (Hanil Scientific Inc., Gyeonggi-do, Korea), followed by staining with Diff-Quik (Thermo Fisher Scientific). Differential counts were performed by counting 300 cells per slide under a light microscope (Nikon, Tokyo, Japan). The BALF supernatants were analyzed for lactate dehydrogenase (LDH) activity, total protein, and proinflammatory cytokines (IL-1β, IL-6, and TNF-α) using an LDH assay kit (Roche, Basel, Switzerland), a bicinchoninic acid protein assay kit (Thermo Fisher Scientific), and DuoSet ELISA kits (R&D Systems, Minneapolis, MN, USA), respectively.

### 2.7 Evaluation of oxidative potential in BALF-derived alveolar macrophages

After cytological analysis of BALF cells, the remaining cells were incubated at a density of 2 × 10^5^ macrophages/mL in 96-well black culture plates (SPL Life Science, Gyeonggi-do, Korea) to evaluate nanoplastics-induced alterations in cellular ROS levels in alveolar macrophages. Because alveolar macrophages are the adherent cell population among BALF cells [20], the cells were incubated for 2 h in a CO_2_ incubator to allow macrophage attachment. After incubation, the supernatants were removed, and the attached cells were washed three times with pre-warmed PBS. The alveolar macrophages were treated with 100 μM DCFH-DA in phenol red-free RPMI-1640 (Sigma-Aldrich, St Louis, MO, USA) for 45 min. Then, cellular ROS were measured at 485/520 fluorescence using a microplate reader (BioTek).

### 2.8 Real-time PCR analysis of NLR family pyrin domain-containing 3 (NLRP3) inflammasome in the lungs

To evaluate the mechanism of the oxidative pathway, gene expression of the NLRP3 inflammasome complex, including caspase-1, apoptosis-associated speck-like protein containing a CARD (ASC), and NLRP3, was measured in alveolar macrophages from BALF at 24 h after a single exposure to 100 μg/mouse. The alveolar macrophages were incubated at a density of 5 × 10^5^ cells/mL in 6-well culture plates (SPL Life Science) for 2 h, then washed with pre-warmed PBS to remove non-attached cells. The alveolar macrophages were treated with 1 mL of TRIzol reagent (Invitrogen, Waltham, MA, USA) for RNA isolation. Total RNA was isolated according to the manufacturer's instructions. The purity and concentration of the extracted RNA were determined using a Nanodrop spectrophotometer (Optizen NanoQ, Mecasys, Daejeon, Republic of Korea). Reverse transcription was carried out using 100 ng/μL of RNA with the High-Capacity cDNA Reverse Transcription Kit (Applied Biosystems, Waltham, MA, USA) following the manufacturer's protocol. Real-time quantitative PCR was performed on a Bio-Rad real-time PCR system (Hercules, CA, USA) using TaqMan™ chemistry (Applied Biosystems). Each 20 μL reaction contained 10 μL of TaqMan™ Universal PCR Master Mix, 1 μL of TaqMan™ Gene Expression Assay, and 9 μL of template cDNA (10 ng/μL). The assays used were GAPDH (Assay ID: Mm99999915_g1), ASC (Assay ID: Mm00445747_g1), caspase-1 (Assay ID: Mm00438023_m1), and NLRP3 (Assay ID: Mm00840904_m1). Cycling conditions were 95 °C for 10 min, followed by 40 cycles of 95 °C for 15 s and 60 °C for 1 min. Ct values were normalized to GAPDH, and relative gene expression levels were calculated using the 2^–ΔΔCt^ method. Four mice were assigned to each group in the evaluation of ROS-related gene expression in alveolar macrophages.

### 2.9 Evaluation of lung inflammation following antioxidant modulation of nanoplastics

N-acetyl-l-cysteine (NAC), a well-established ROS scavenger, was used to evaluate the contribution of the oxidative potential of nanoplastics to lung inflammation. Briefly, UFPS (2 mg/mL) was incubated with NAC (10 mM) for 1 h, as previously described [21]. The mixture was centrifuged at 21,000 × *g* for 30 min, after which the supernatant was discarded, and the pellet was resuspended in DW. This washing procedure was repeated three times. Finally, the particle pellets were resuspended in PBS, assessed for oxidative potential using the DCFH-DA assay and for changes in functional groups using XPS analysis, and instilled into mice at 100 μg/mouse to compare lung inflammatory responses induced by UFPS with and without NAC treatment, following the identical protocol described above. This study was also measured on NLRP3 inflammasome gene expression using the above-described method. Four mice were used for the NAC-treated UFPS group, while the comparison data for UFPS alone were obtained from the corresponding group in the original experiment described above.

### 2.10 Evaluation of the pulmonary clearance pattern of test nanoplastics

To evaluate the pulmonary clearance kinetics of test nanoplastics, lung burden analysis was performed at multiple time points up to 3 months after a single exposure, using a previously described method [22,23]. Briefly, whole lungs were collected at 0, 1, 7, 14, 28, 60, and 90 days post-exposure, dried in an oven at 56 °C for 2 days, and then the dried tissue was weighed. To assess whole-lung particle retention, dried lung tissue was subjected to a two-step proteinase K (PK) digestion procedure to isolate retained nanoplastics. For the first digestion, PK buffer was added at 1 mL of PK buffer (50 mM Tris-HCl, 10 mM CaCl_2_, and 100 μg/mL proteinase K) per 20 mg of dried lung tissue and incubated at 56 °C for 24 h, followed by centrifugation at 21,000 × *g* for 30 min to collect the pellet containing the particles. To further remove residual tissue components, the pellets were resuspended in 1 mL of fresh PK buffer and subjected to a second digestion at 56 °C for an additional 24 h, followed by a second centrifugation at 21,000 × *g* for 30 min. The final pellets were resuspended in 1 mL of DW for subsequent lung burden analysis. The collected suspensions containing nanoplastics were analyzed spectrophotometrically at 750 nm, a wavelength selected to minimize interference from biomolecules [21]. Nanoplastics concentrations were quantified using a standard calibration curve generated from known concentrations of the corresponding test nanoplastics based on absorbance at 750 nm. Prior to application of this method, the accuracy and recovery of the tissue digestion and quantification procedure were evaluated to confirm methodological reliability.

### 2.11 Wound healing assay for migration test in differentiated THP-1 cells

To evaluate macrophage cell migration following nanoplastic treatment, THP-1 cells were used in this study. Furthermore, the test nanoplastics were dispersed in FBS and then prepared using the same methods as in the *in vivo* study, but the final dispersion medium was Roswell Park Memorial Institute-1640 (Welgene, Gyeongsangbuk-do, Korea) without FBS. Briefly, the THP-1 cell line was purchased from the American Type Culture Collection (ATCC; Manassas, VA, USA). The THP-1 cells were treated with phorbol myristate acetate (PMA; Sigma-Aldrich) at 10 ng/mL for 48 h in RPMI-1640 containing 10% FBS to differentiate into macrophage-like cells. The cells were seeded at 5 × 10^5^ cells/mL in 96-well cell culture plates (SPL Life Science). After 48 h of incubation, the cells were washed 3 times with pre-warmed PBS, then treated with 50 μg/mL of all test nanoplastics in RPMI-1640 without FBS, the selected concentration, as no toxicity was observed in THP-1 cells according to our previously described method [10]. After 6 h of incubation, the supernatants were removed, and the cells were washed with pre-warmed PBS for 3 times. Then, the cells were added to new RPMI-1640 without FBS. The cells were incubated for 6 h, and the cell migration was confirmed at 6, 12, 24, and 48 h using light microscopy.

### 2.12 Statistical analysis

Data from repeated measurements are presented as the mean ± standard deviation (SD) (*n* = 4). Statistical analyses and figure generation were performed using GraphPad Prism (version 10.6.0; GraphPad Software; La Jolla, CA, USA). Group differences were evaluated using one-way analysis of variance (ANOVA) followed by Tukey's post hoc test for multiple comparisons. The Mann-Whitney *U* test was used to compare UFPS with or without NAC. Correlation analyses were performed using Spearman's correlation test. A p-value <0.05 was considered statistically significant.

## 3 Results

### 3.1 Physicochemical properties of test nanoplastics

**Fig. 1:**
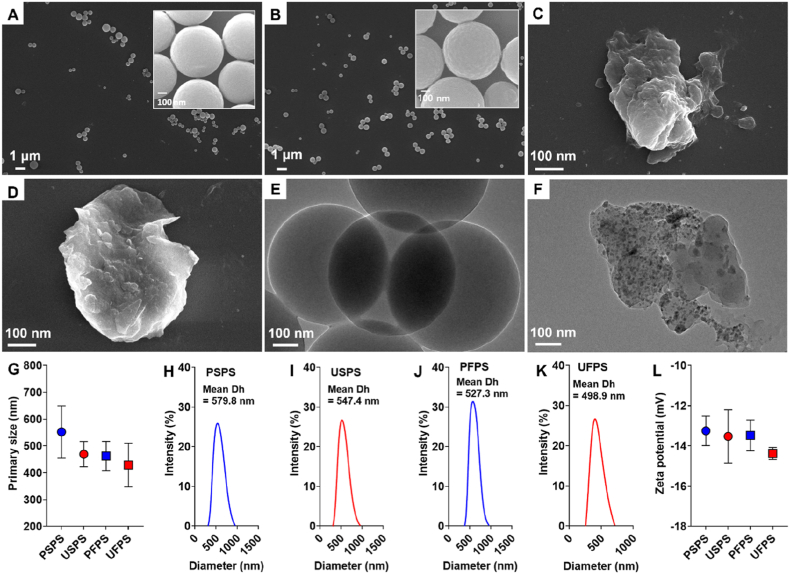
Physicochemical properties of test nanoplastics. Scanning electron microscopy (SEM) images of (A) pristine spherical polystyrene (PSPS), (B) UV-irradiated spherical polystyrene (USPS), (C) pristine fragmented polystyrene (PFPS), and (D) UV-irradiated fragmented polystyrene (UFPS). Transmission electron microscopy (TEM) images of (E) PSPS and (F) PFPS. (G) Primary particle size (nm) of test nanoplastics. Hydrodynamic diameter (Dh) of (H) PSPS, (I) USPS, (J) PFPS, and (K) UFPS in phosphate-buffered saline (PBS). The mean hydrodynamic diameter for each particle type was labeled in figure. (L) Zeta potential of test nanoplastics in PBS. Data are presented as mean ± SD (*n* = 300 per group for G and *n* = 4 per group for H–L).

To characterize differences between primary and secondary nanoplastics, various physicochemical properties were measured. The PSPS and USPS nanoplastics exhibited spherical morphology, whereas the PFPS and UFPS nanoplastics showed fragmented and irregular morphologies in SEM and TEM images (Fig. 1A–F). The primary size of test nanoplastics was 429–552 nm (Fig. 1G). The BET values for PSPS and PFPS were 14.17 and 15.26 m^2^/g, respectively, while those for USPS and UFPS decreased to 13.85 and 11.98 m^2^/g, respectively (Table S1). The hydrodynamic diameter and zeta potential were measured in PBS after dispersing the test particles, as these parameters reflect their dispersion stability and potential particle–biomolecule interactions. The hydrodynamic diameters ranged from 499 to 580 nm in PBS across particle types (Fig. 1H–K and Table S1). The zeta potential of all test nanoplastics was negative in PBS (Fig. 1L and Table S1).

### 3.2 UV-induced surface oxidation of test particles

**Fig. 2:**
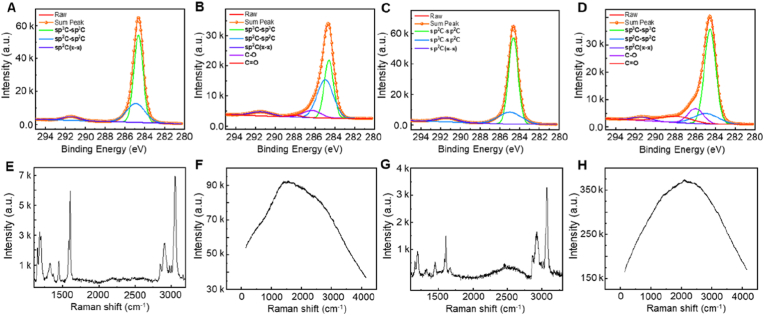
Spectroscopic evidence of UV-induced surface oxidation in test nanoplastics. X-ray photoelectron spectroscopy (XPS) analysis of (A) pristine spherical polystyrene (PSPS), (B) UV-irradiated spherical polystyrene (USPS), (C) pristine fragmented polystyrene (PFPS), and (D) UV-irradiated fragmented polystyrene (UFPS). Raman spectroscopy of (E) PSPS, (F) USPS, (G) PFPS, and (H) UFPS.

XPS analysis revealed distinct changes in the surface chemistry of polystyrene following UV exposure, characterized by a decreased contribution of C–C bonds and a concomitant increase in oxygen-containing functional groups (C–O and C=O) in both fragmented (PFPS vs. UFPS) and spherical particles (PSPS vs. USPS) (Fig. 2A–D and Table S2). Confocal Raman spectroscopy at 532 nm showed that PSPS and PFPS exhibited largely similar spectral features, characterized by typical polystyrene bands, including benzene ring deformation, out-of-plane C–H deformation, C–C vibration regions, and CH_2_ and CH_3_ stretching modes. Notably, a C=C stretching peak at 1671 cm^−1^ was observed only in PFPS (Fig. 2E and G). In contrast, UV-irradiated particles (i.e., USPS and UFPS) exhibited strong fluorescence under 532 nm excitation (Fig. 2F and H).

### 3.3 Comparative acute lung inflammatory potential of test nanoplastics

#### 3.3.1 Cytological analysis of BALF

**Fig. 3:**
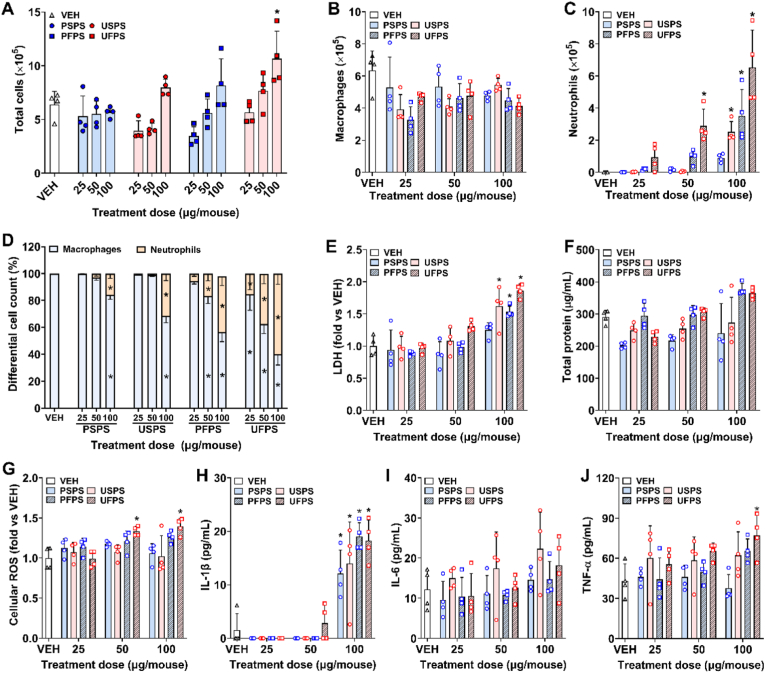
Comparative acute lung inflammatory potential of test nanoplastics. Acute lung inflammation was assessed by bronchoalveolar lavage fluid (BALF) analysis 1 day after a single pharyngeal aspiration of nanoplastics in mice at doses of 25, 50, and 100 μg/mouse. (A–D) Cytological analysis of BALF showing (A) total cell counts, (B) alveolar macrophage counts, (C) neutrophil counts, and (D) differential cell percentages. (E–F) Biochemical analysis of BALF, including (E) lactate dehydrogenase (LDH) levels and (F) total protein levels. (G) Oxidative potential of alveolar macrophages isolated from BALF, measured using the DCFH-DA assay. (H–J) Levels of pro-inflammatory cytokines in BALF, including (H) interleukin (IL)-1β, (I) IL-6, and (J) tumor necrosis factor (TNF)-α. PSPS, pristine spherical polystyrene; USPS, UV-irradiated spherical polystyrene; PFPS, pristine fragmented polystyrene; UFPS, UV-irradiated fragmented polystyrene. Data are presented as mean ± SD (*n* = 4). ∗*p* < 0.05 indicates a statistically significant difference compared with the vehicle (VEH) control group, as determined by one-way ANOVA followed by Tukey's post hoc test.

The acute pulmonary inflammatory potential of the test particles was assessed using BALF analysis 24 h after pharyngeal aspiration in mice. Total cell counts in BALF increased in a dose-dependent manner for all particle types except PSPS, which showed relatively constant levels; statistically significant increases compared with the VEH group were observed only at the high dose of UFPS (Fig. 3A). Differential cell counts showed that alveolar macrophage numbers did not differ significantly from those in the VEH group (Fig. 3B). In contrast, neutrophil counts increased in a dose-dependent manner for all tested particles, with the magnitude of the increase following the order UFPS > PFPS > USPS > PSPS (Fig. 3C). Consistent with the increased neutrophil counts, the percentage of alveolar macrophages decreased in a dose-dependent manner, reaching statistical significance at the high dose for all test particles compared with the VEH group, although absolute macrophage numbers did not change significantly (Fig. 3D). The percentage of neutrophils increased significantly across all test particles relative to the VEH group, with the magnitude of the increase following the order UFPS > PFPS > USPS > PSPS (Fig. 3D). The detailed differences in neutrophilic inflammatory potency among the test particles are summarized in Table S3. When interpreted in terms of UV exposure at the highest tested dose, UFPS induced a 1.9-fold greater neutrophilic inflammatory response than PFPS, whereas USPS elicited a 2.8-fold greater neutrophilic response than PSPS. By contrast, when classified by particle morphology, UFPS induced a 2.6-fold greater neutrophilic inflammatory response than USPS, and PFPS induced a 3.9-fold greater response than PSPS.

#### 3.3.2 BALF LDH and total protein levels and oxidative potential of alveolar macrophages

LDH levels in BALF showed a weaker response than neutrophil counts but followed a similar trend (UFPS > PFPS > USPS > PSPS), and statistical significance increases were observed only at the high dose for UFPS, PFPS, and USPS, but not for PSPS, compared with the VEH group (Fig. 3E). Although BALF total protein levels showed a modest, dose-dependent increase, differences among particle types were minimal, and no statistically significant changes were observed at any dose compared with the VEH group; the overall response was weaker than that of LDH (Fig. 3F). Cellular ROS levels remained largely unchanged across doses in the PSPS-, USPS-, and PFPS-treated groups. In contrast, UFPS induced a modest but dose-dependent increase in cellular ROS levels, reaching statistical significance at the mid and high doses compared with the VEH group. At the highest tested dose, relative cellular ROS levels followed the order UFPS > PFPS > USPS ≈ PSPS (Fig. 3G). Furthermore, at 24 h after exposure, NLRP3 expression tended to increase more clearly than caspase-1 or ASC in alveolar macrophages, especially in the fragmented particle groups. This result suggests a greater association of fragmented nanoplastics with early inflammasome-related transcriptional responses (Fig. S1, see Supporting Information).

#### 3.3.3 Proinflammatory cytokines in BALF

Among the pro-inflammatory cytokines tested (IL-1β, IL-6, and TNF-α), only IL-1β and TNF-α showed significant increases, whereas IL-6 levels remained unchanged following nanoplastics treatment (Fig. 3H–J). IL-1β levels exhibited a distinct response pattern, showing minimal changes at low and mid doses but a significant increase only at the high dose across all tested particles compared with the VEH group; however, no statistically significant differences were observed among particle types (Fig. 3H). TNF-α levels showed a modest increase only in the high-dose UFPS group compared with the VEH group, while spherical particles (PSPS and USPS) and PFPS maintained relatively constant levels; nevertheless, no significant inter-particle differences were detected (Fig. 3J).

### 3.4 Effect of particle-derived oxidative potential on cellular and *in vivo* toxicity endpoints

To assess the impact of particle-derived oxidative potential on cellular and pulmonary inflammatory responses, correlation analyses were conducted between the intrinsic ROS-generating capacity of nanoplastics, cellular ROS levels, and multiple *in vivo* lung inflammation parameters. Here, intrinsic oxidative potential refers to the ability of particles to generate ROS directly, independent of cellular processes.

#### 3.4.1 The comparative intrinsic oxidative potential of test particles

**Fig. 4:**
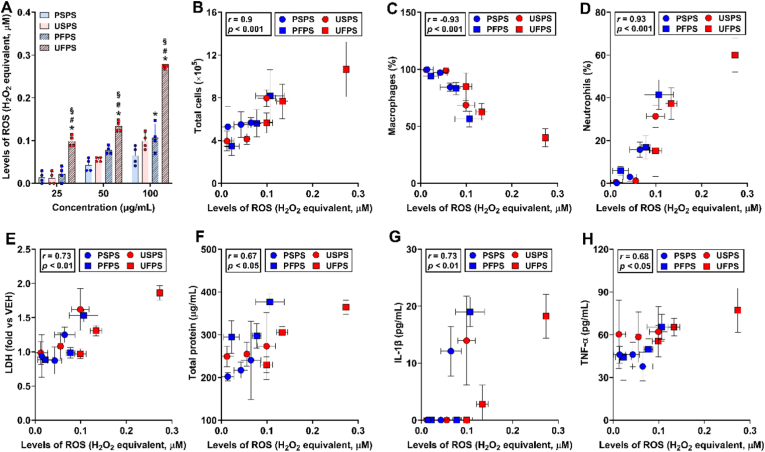
Oxidative potential of test nanoplastics and its correlation with the lung inflammation parameters. (A) Intrinsic reactive oxygen species (ROS)–generating capacity of the test particles. ∗*p* < 0.05 indicates significant differences compared to the vehicle control group, as determined by one-way ANOVA followed by Tukey's post hoc test. (B–H) Spearman's correlation analyses between intrinsic ROS-generating capacity of nanoplastics and pulmonary inflammatory parameters in bronchoalveolar lavage fluid (BALF) 1 day after a single pharyngeal aspiration in mice: (B) total cell counts, (C) percentage of alveolar macrophages, (D) percentage of neutrophils, (E) lactate dehydrogenase (LDH) levels, (F) total protein levels, (G) interleukin (IL)-1β levels, and (H) tumor necrosis factor (TNF)-α levels. The values shown in each panel represent the Spearman correlation coefficient (*r*) and its corresponding *p*-value. PSPS, pristine spherical polystyrene; USPS, UV-irradiated spherical polystyrene; PFPS, pristine fragmented polystyrene; UFPS, UV-irradiated fragmented polystyrene. Data are presented as mean ± standard deviation (SD) (*n* = 4). Statistical significance was determined by one-way ANOVA followed by Tukey's post hoc test (∗*p* < 0.05 vs. PSPS; ^#^*p* < 0.05 vs. USPS; ^§^*p* < 0.05 vs. PFPS).

Based on H_2_O_2_-equivalent oxidative values, all test particles exhibited a concentration-dependent increase in intrinsic ROS generation (Fig. 4A). Across particle types, intrinsic ROS-generating capacity followed the order UFPS > PFPS > USPS > PSPS, consistent with the greater extent of surface oxidation observed by XPS analysis in UFPS. Notably, the UV-induced enhancement of particle-associated oxidative reactivity was more pronounced in fragmented particles than in spherical particles.

#### 3.4.2 Association between intrinsic oxidative potential and cellular ROS levels

The intrinsic ROS-generating capacity of the test particles showed poor correlation with cellular ROS levels in alveolar macrophages collected from BALF 24 h after nanoplastics exposure (Fig. S2, see Supporting Information). Likewise, cellular ROS levels showed no significant correlations with pulmonary toxicity endpoints in BALF at 24 h post-exposure.

#### 3.4.3 Association between intrinsic oxidative potential and inflammatory responses

The intrinsic oxidative potential showed strong correlations with most pulmonary toxicity endpoints in BALF at 24 h post-exposure. Specifically, intrinsic ROS-generating capacity was significantly correlated with total cell counts and the relative proportions of macrophages and neutrophils (Spearman *r* = 0.90, *p* < 0.001 for total cell counts; *r* = −0.93, *p* < 0.001 for macrophage percentage; *r* = 0.93, *p* < 0.001 for neutrophil percentage) (Fig. 4B–D). Absolute neutrophil counts also showed good correlations with intrinsic ROS levels (Fig. S2, see Supporting Information). Notably, although LDH and total protein levels in BALF exhibited only modest changes, both parameters showed significant positive correlations with intrinsic ROS-generating capacity (Fig. 4E and F). In addition, IL-1β and TNF-α levels were positively correlated with intrinsic ROS levels (Fig. 4G and H), whereas no significant correlation was observed between intrinsic ROS generation and IL-6 levels in BALF (Fig. S2, see Supporting Information).

### 3.5 Modulation of intrinsic ROS-associated pulmonary responses by NAC

**Fig. 5:**
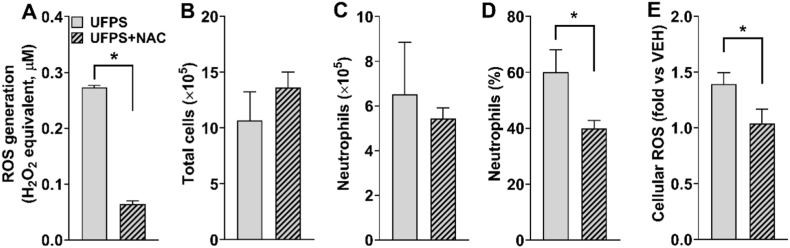
Effects of N-acetyl cysteine (NAC) pretreatment on oxidative stress and pulmonary inflammation induced by UFPS. UFPS was pretreated with NAC (UFPS vs. NAC-pretreated UFPS), and acute lung inflammation was evaluated by bronchoalveolar lavage fluid (BALF) analysis 1 day after a single pharyngeal aspiration in mice at a dose of 100 μg/mouse. (A) Intrinsic reactive oxygen species (ROS)–generating capacity of UFPS with or without NAC pretreatment. (B–D) Cytological analysis of BALF showing (B) total cell counts, (C) absolute neutrophil counts, and (D) percentage of neutrophils. (E) Cellular ROS levels were measured in alveolar macrophages isolated from BALF. UFPS, UV-irradiated fragmented polystyrene. Data are presented as mean ± SD (*n* = 4). ∗*p* < 0.05 indicates a statistically significant difference between groups, as determined by the Mann-Whitney *U* test.

NAC-pretreated UFPS exhibited a significantly reduced intrinsic ROS-generating capacity compared with untreated UFPS (Fig. 5A). Following pharyngeal aspiration in mice, NAC-pretreated UFPS induced significantly lower neutrophilic inflammatory responses compared with the UFPS group, as reflected by reduced neutrophil percentages in BALF (60% to 40%) and decreased absolute neutrophil counts (6.5 × 10^5^ cells to 5.4 × 10^5^ cells). In contrast, total cell counts did not differ significantly between the two groups (Fig. 5B–D). In addition, cellular ROS levels were significantly reduced by NAC pretreatment (1.39-fold to 1.04-fold) (Fig. 5E). Furthermore, the ASC and NLRP3 gene expression within the NLRP3 inflammasome complex was significantly decreased by NAC treatment (Fig. S3, see Supporting Information). To investigate whether pretreatment with NAC altered the surface characteristics of UFPS, XPS analysis was conducted. Compared with untreated UFPS, NAC-pretreated UFPS showed a reduced relative abundance of C=O groups and an increased relative abundance of C–O groups (Table S2, see Supporting Information).

### 3.6 Recovery of lung inflammation 1 month after a single exposure

**Fig. 6:**
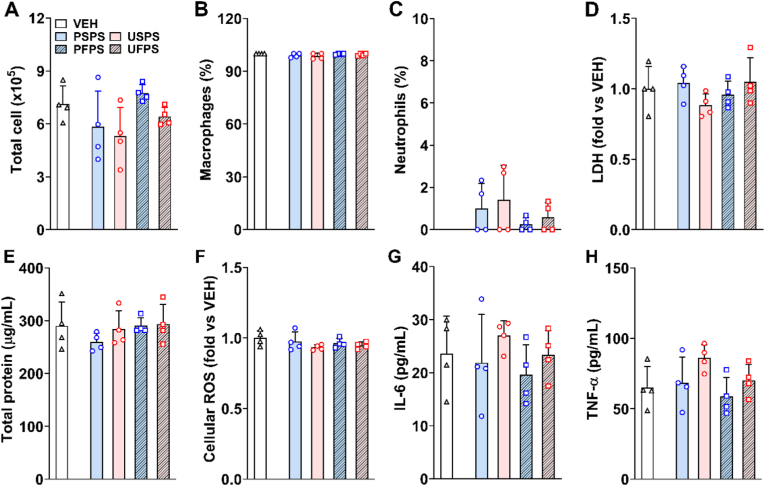
Recovery of acute pulmonary inflammation at 1 month after a single nanoplastics exposure. To evaluate the reversibility of acute lung inflammation observed at 24 h post-exposure, pulmonary inflammatory responses were assessed by bronchoalveolar lavage fluid (BALF) analysis 1 month after a single pharyngeal aspiration of nanoplastics in mice at a dose of 100 μg/mouse. (A–E) BALF inflammatory parameters, including (A) total cell counts, (B) percentage of macrophages, (C) percentage of neutrophils, (D) lactate dehydrogenase (LDH) levels, and (E) total protein levels. (F) Cellular reactive oxygen species (ROS) levels in alveolar macrophages isolated from BALF. (G–H) Levels of pro-inflammatory cytokines in BALF, including (G) interleukin (IL)-6 and (H) tumor necrosis factor (TNF)-α. PSPS, pristine spherical polystyrene; USPS, UV-irradiated spherical polystyrene; PFPS, pristine fragmented polystyrene; UFPS, UV-irradiated fragmented polystyrene. Data are presented as mean ± SD (*n* = 4). ∗*p* < 0.05 indicates significant differences compared to the vehicle control group, as determined by one-way ANOVA followed by Tukey's post hoc test.

To assess the reversibility of nanoplastics-induced pulmonary inflammation, BALF analysis was performed 1 month after a single pharyngeal aspiration at a dose of 100 μg/mouse. At this time point, all pulmonary inflammatory parameters, including BALF cell counts, LDH and total protein levels, cellular ROS levels in alveolar macrophages isolated from BALF, and pro-inflammatory cytokines [IL-1β (all below the detection limit), IL-6, and TNF-α], in nanoplastics-treated groups had returned to levels comparable to those in the control group (Fig. 6).

### 3.7 Lung clearance kinetics of nanoplastics

**Fig. 7:**
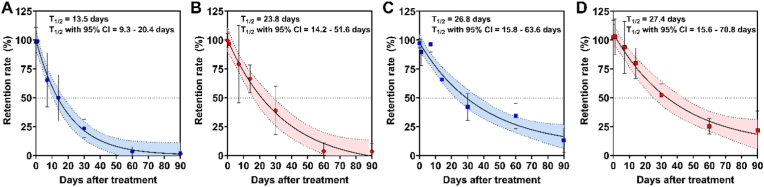
Lung clearance kinetics of test nanoplastics in mice. Mice were exposed to test particles by pharyngeal aspiration at a dose of 25 μg/mouse, and lung burden mass was quantified at 0, 1, 7, 14, 28, 60, and 90 days post-exposure. (A-D) Lung clearance kinetics of (A) PSPS, (B) USPS, (C) PFPS, and (D) UFPS. PSPS, pristine spherical polystyrene; USPS, UV-irradiated spherical polystyrene; PFPS, pristine fragmented polystyrene; UFPS, UV-irradiated fragmented polystyrene. Data are presented as mean ± SD (*n* = 4). Nonlinear regression using a one-phase exponential decay model was applied to estimate lung clearance half-lives (T_1/2_) and their corresponding 95% confidence intervals (CIs).

To evaluate the effects of particle morphology and surface oxidation on pulmonary clearance, lung clearance patterns were assessed for up to 6 months using four types of polystyrene (PS) particles (spherical vs. fragmented; pristine vs. UV-irradiated). The newly developed lung burden analysis method, based on PK tissue digestion followed by UV–Vis spectrophotometric quantification, demonstrated that all nanoplastics could be quantified using standard curve fitting with excellent linearity (*r* = 0.999, *p* < 0.001) (Fig. S4A–D, see Supporting Information). In addition, recovery experiments using lung tissue homogenates spiked with test particles showed recovery rates exceeding 92% for all nanoplastics (Fig. S4E, see Supporting Information). Analysis of lung clearance patterns showed that fragmented particles exhibited longer lung retention than spherical particles, whereas UV irradiation had little to no effect on lung clearance (Fig. 7). Spherical nanoplastics, regardless of UV irradiation, showed a marked reduction beginning at day 7 post-exposure, with approximately 30–40% remaining at 1 month and most particles being cleared by 2–3 months (Fig. 7A and B). The lung clearance half-lives (T_1/2_) of PSPS and USPS were estimated to be 13.5 and 23.8 days, respectively, with corresponding 95% confidence intervals (CIs) of 9.3–20.4 days for PSPS and 14.2–51.6 days for USPS. In contrast, fragmented nanoplastics exhibited a delayed lung clearance pattern, with a gradual decrease beginning at day 14 and persistence up to 3 months, during which approximately 13–22% of the particles remained in the lung (Fig. 7C and D). The lung clearance T_1/2_ of PFPS and UFPS were estimated to be 26.8 and 27.4 days, respectively, with corresponding 95% CIs of 15.8–63.6 days for PFPS and 15.6–70.8 days for UFPS. Moreover, consistent with the delayed lung clearance, macrophages exposed to fragmented nanoplastics exhibited lower migratory capacity than those exposed to spherical nanoplastics (Fig. S5, see Supporting Information).

## 4 Discussion

Respiratory exposure to airborne nanoplastics is an emerging environmental health concern; however, their pulmonary behavior and toxicological relevance remain incompletely understood under inhalation-relevant conditions [24,25]. Overall, our results demonstrate that nanoplastics-induced pulmonary toxicity is markedly amplified by particle fragmentation and environmental aging. Fragmented and UV-aged PS nanoplastics elicited more pronounced acute lung inflammation than pristine spherical particles, and these heightened inflammatory responses were closely associated with increased surface oxidation and a concomitant increase in the particles’ intrinsic oxidative reactivity. In contrast, pulmonary clearance kinetics were governed primarily by particle morphology rather than oxidative reactivity, with fragmented particles exhibiting delayed lung clearance relative to spherical particles, irrespective of their UV-induced oxidation state. Collectively, surface chemistry–driven oxidative stress emerged as a key determinant of inflammatory potency, with particle morphology contributing to this process through its close association with surface oxidation and intrinsic oxidative reactivity, while exerting a dominant influence on pulmonary persistence. These results highlight the critical role of environmental transformation and physicochemical properties in understanding nanoplastics-induced pulmonary toxicity.

When evaluating the toxicity of environmentally relevant nanoplastics, particles produced via top-down fragmentation should be preferentially considered. Fragmented nanoplastics possess a higher specific surface area and a greater density of reactive surface sites, such as structural defects and exposed functional groups, which facilitate surface-driven electron transfer reactions and enhance ROS generation [26,27]. Enhanced surface reactivity induces early cytotoxic stress in exposed cells, leading to membrane destabilization and cellular dysfunction. This cytotoxicity can involve multiple modes of cell injury, including direct particle-induced membrane damage, necrotic cell death, and stress-associated injury pathways [28,29]. Importantly, such initial cytotoxic stress often precedes or occurs concurrently with the recruitment and activation of alveolar macrophages, which act as the primary immune sentinels that recognize, engulf, and attempt to neutralize inhaled foreign materials [30,31]. In this context, enhanced ROS generation resulting from increased surface reactivity can further exacerbate oxidative damage to cellular membranes and intracellular organelles, thereby amplifying cytotoxic responses and promoting downstream inflammatory signaling [32]. Collectively, these mechanisms suggest that particle shape–dependent increases in surface reactivity and ROS generation contribute to early cytotoxic stress, which sets the stage for subsequent macrophage-driven inflammatory responses and ultimately influences the overall toxicological outcome in the lung.

The oxidative potential of nanoplastics increases through environmental aging processes, including UV irradiation and weathering, which introduce oxygen-containing functional groups (e.g., C=O, C–O, and –OH) and structural defects that facilitate electron-transfer reactions at the particle surface [33,34]. Consistent with this, UV-aged and fragmented PS nanoplastics exhibited enhanced ROS-generating potential, attributable to increased surface oxidation, disruption of π–π∗ structures, and an enlarged specific surface area [33,35]. In addition, residual metal catalysts or inorganic additives (e.g., TiO_2_, Fe-containing pigments) embedded in plastics can further promote ROS formation through photocatalytic or Fenton-like reactions [36]. Upon cellular internalization, these surface-driven effects are amplified by biological mechanisms, including lysosomal destabilization, mitochondrial dysfunction, and activation of nicotinamide adenine dinucleotide phosphate oxidases, thereby elevating intracellular ROS production and oxidative stress responses [37,38]. However, the weak correlation observed in this study between particle-derived intrinsic ROS generation and cellular ROS levels in alveolar macrophages suggests that elevated oxidative potential does not necessarily result in sustained intracellular ROS accumulation. Instead, particle-generated ROS may act as upstream triggers of inflammatory signaling pathways, such as IL-1β production and neutrophil recruitment, rather than directly driving intracellular oxidative stress [39]. This dissociation between particle-derived ROS and cellular ROS responses is consistent with the observed cytokine patterns and indicates that nanoplastics-induced inflammation may proceed via ROS-sensitive signaling mechanisms without an overt elevation in intracellular ROS.

In this context, enhanced surface-driven oxidative reactivity may not only increase direct cytotoxic stress but also promote downstream innate inflammatory signaling in macrophages. Particle-associated ROS can contribute to the priming and activation of the NLRP3 inflammasome, which is more directly linked to caspase-1 activation and IL-1β-associated responses, whereas ROS-sensitive MAPK/NF-κB signaling may regulate broader inflammatory mediators, such as TNF-α and IL-6 [21,40,41]. Previous studies have shown that polystyrene nanoplastics induce ROS-dependent MAPK/NF-κB activation with increased TNF-α and IL-6 expression in macrophages, and that polystyrene fragments in mouse lungs activate TLR4/NF-κB/NLRP3-associated inflammatory signaling with increased NLRP3 inflammasome components [11,40]. In the present study, however, the inflammatory profile appeared to be more prominently associated with inflammasome-related responses than with a strong IL-6-dominant pattern. This difference may reflect the particle-specific nature of the response, in which environmentally transformed fragmented nanoplastics preferentially impose oxidative and cellular stress on macrophages, thereby favoring inflammasome-associated innate immune signaling over a broader cytokine-dominant response [21,41]. Together, these observations indicate that the pulmonary effects of environmentally transformed fragmented nanoplastics are more closely associated with oxidative stress–inflammasome-related innate immune responses than with a broad cytokine-dominant inflammatory pattern.

NAC is a thiol-containing antioxidant widely used as a ROS scavenger and glutathione precursor [42]. However, NAC may not act solely through intracellular ROS scavenging, because its nucleophilic thiol group can also directly react with electrophilic or oxidized chemical species [43]. This point may be particularly relevant for environmentally transformed plastic particles, as UV aging of polystyrene has been reported to induce oxygen-containing functional groups, surface roughening, and physical defects such as cracks, thereby increasing surface reactivity [44,45]. The significant attenuation of particle-induced toxic responses by NAC observed in this study further supports the involvement of reactive surface features—such as surface defect sites and oxygen-containing functional groups—in promoting ROS generation at the particle interface [46,47]. At the same time, these findings should be interpreted with caution, because the protective effects of NAC may reflect not only intracellular ROS scavenging but also possible direct interactions with oxidized particle surfaces. XPS analysis further showed that NAC treatment altered the surface chemical composition of UFPS, as reflected by a decrease in the relative C=O contribution and an increase in the C–O component. However, it remains unclear whether these changes are directly linked to the particles' reduced ROS-generating capacity, since the specific contribution of individual oxygen-containing functional groups to particle-associated oxidative reactivity has not been fully resolved. Therefore, additional studies are needed to clarify how NAC interacts with microplastic surfaces and whether such interactions directly influence particle-associated ROS generation and downstream toxic responses.

Beyond differences in cellular uptake, particle shape emerged as a critical determinant of pulmonary retention under the experimental conditions of this study. By employing PS nanoplastics with comparable particle sizes that differed only in morphology and UV-induced surface aging, we excluded particle size as a confounding factor influencing cellular uptake. Within this size-controlled context, our findings indicate that particle morphology plays a dominant role in determining pulmonary persistence. Particle shape has been shown to influence cellular motility, as spherical nanoparticles tend to preserve normal cytoskeletal organization, whereas irregular or elongated particles disrupt actin dynamics and impair cell migration [[48], [49], [50]]. Similarly, sharp or irregular particles can perturb membrane curvature sensing and focal adhesion turnover, ultimately slowing directional cell movement [51,52]. In macrophages, elongated or fiber-like particles frequently induce frustrated phagocytosis, a process that not only limits particle internalization but also reduces macrophage motility, thereby delaying particle transport and clearance from lung tissue [53]. Consistent with these mechanisms, our results demonstrate that particle geometry critically dictates pulmonary clearance dynamics, with spherical primary nanoplastics being removed more efficiently, whereas fragmented secondary nanoplastics exhibit prolonged lung retention due to impaired migratory and phagocytic activity.

Inhaled nanoplastics with diameters below 1 μm can be cleared from the respiratory tract via mucociliary transport or macrophage-driven phagocytosis, while a fraction may translocate to secondary organs [54]. In the present study, pulmonary clearance kinetics were evaluated using a single bolus dose of 25 μg per mouse, which is not generally considered sufficient to induce macrophage overload. Notably, differences in pulmonary clearance persisted under these conditions, suggesting that impaired clearance cannot be solely attributed to particle overload. Nevertheless, it is well established that repeated inhalation exposure to particles can progressively increase lung burden, and the accumulated particle load exceeds the phagocytic capacity of alveolar macrophages, leading to impaired macrophage function and delayed pulmonary clearance [55]. In real-world exposure scenarios, where inhalation is likely to be repeated over time, morphology-driven effects on pulmonary retention may therefore be further amplified rather than newly triggered by particle overload.

Because this study was a single-exposure experiment conducted under controlled conditions, the results do not fully capture the cumulative lung burden and persistent pulmonary responses that may arise during repeated or chronic inhalation exposure [56,57]. In addition, the pharyngeal aspiration model used here does not fully reproduce the aerosol behavior and regional deposition patterns of real-world human inhalation, and therefore, direct quantitative extrapolation to humans should be made with caution [58]. The use of only female BALB/c mice is also a limitation, as sex-dependent differences in pulmonary inflammatory responses have been reported in rodents [18]. This design was chosen to maintain consistency with our established pharyngeal aspiration-based pulmonary exposure and to enable controlled comparisons of particle morphology and UV aging while minimizing sex- and age-related variability. Future studies that include both male and female animals are warranted to determine whether the inflammatory and clearance responses observed here are sex-dependent. Despite these limitations, the present model remains useful for comparing relative differences in acute pulmonary toxicity and clearance associated with particle morphology and UV aging. Moreover, although recent pulmonary toxicity studies have increasingly used inhalation or repeated airway exposure models, most have focused on PS, PP, and PE. In this regard, the present study adds value by examining EPS-derived particles, a comparatively less investigated expanded polymer material with distinct physicochemical characteristics.

## 5 Conclusion

This study demonstrates that secondary nanoplastics generated through environmental fragmentation and UV-induced surface aging pose a greater pulmonary hazard than pristine nanoplastics following inhalation exposure. Under size-controlled experimental conditions, in which four PS nanoplastics samples with comparable particle sizes but distinct morphologies and surface oxidation states were evaluated, differences in pulmonary toxicity were primarily attributable to particle morphology and surface-driven oxidative reactivity, whereas particle geometry played a key role in determining pulmonary clearance. Overall, this study demonstrates that environmentally transformed nanoplastics exhibit greater pulmonary toxicity than pristine particles, underscoring the need for further studies to elucidate the health implications of secondary nanoplastics.

## CRediT authorship contribution statement

**Soyeon Jeon:** Writing – original draft, Investigation, Formal analysis, Conceptualization. **Jun Hui Jeon:** Resources, Investigation, Formal analysis. **Gyuri Kim:** Validation, Investigation. **Sung Ik Yang:** Writing – review & editing, Supervision, Project administration. **Wan-Seob Cho:** Writing – review & editing, Writing – original draft, Supervision, Project administration.

## Ethics approval and consent to participate

Not applicable.

## Consent for publication

Not applicable.

## Declaration of competing interest

The authors declare that they have no known competing financial interests or personal relationships that could have appeared to influence the work reported in this paper.

## Data Availability

Data will be made available on request.

## References

[1] Prata J.C. (2018). Airborne microplastics: consequences to human health?. Environ. Pollut..

[2] Zhang Y.L., Kang S.C., Allen S., Allen D., Gao T.G., Sillanpää M. (2020). Atmospheric microplastics: a review on current status and perspectives. Earth Sci. Rev..

[3] Vogel A., Tentschert J., Pieters R., Bennet F., Dirven H., van den Berg A., Lenssen E., Rietdijk M., Brossell D., Haase A. (2024). Towards a risk assessment framework for micro- and nanoplastic particles for human health. Part. Fibre Toxicol..

[4] Turner A. (2020). Foamed polystyrene in the marine environment: sources, additives, transport, behavior, and impacts. Environ. Sci. Technol..

[5] Lambert S., Wagner M. (2016). Characterisation of nanoplastics during the degradation of polystyrene. Chemosphere.

[6] Alimi O.S., Claveau-Mallet D., Kurusu R.S., Lapointe M., Bayen S., Tufenkji N. (2022). Weathering pathways and protocols for environmentally relevant microplastics and nanoplastics: what are we missing?. J. Hazard Mater..

[7] Koelmans A.A., Mohamed Nor N.H., Hermsen E., Kooi M., Mintenig S.M., De France J. (2019). Microplastics in freshwaters and drinking water: critical review and assessment of data quality. Water Res..

[8] Chan H.H., Not C. (2023). Variations in the spatial distribution of expanded polystyrene marine debris: are Asian's coastlines more affected?. Environ. Adv..

[9] Gou Z., Wu H., Li S., Liu Z., Zhang Y. (2024). Airborne micro- and nanoplastics: emerging causes of respiratory diseases. Part. Fibre Toxicol..

[10] Jeon S., Jeon J.H., Jeong J., Kim G., Lee S., Kim S., Maruthupandy M., Lee K., Yang S.I., Cho W.S. (2023). Size- and oxidative potential-dependent toxicity of environmentally relevant expanded polystyrene styrofoam microplastics to macrophages. J. Hazard Mater..

[11] Woo J.H., Seo H.J., Lee J.Y., Lee I., Jeon K., Kim B., Lee K. (2023). Polypropylene nanoplastic exposure leads to lung inflammation through p38-mediated NF-kappaB pathway due to mitochondrial damage. Part. Fibre Toxicol..

[12] Deng J., Ibrahim M.S., Tan L.Y., Yeo X.Y., Lee Y.A., Park S.J., Wustefeld T., Park J.W., Jung S., Cho N.J. (2022). Microplastics released from food containers can suppress lysosomal activity in mouse macrophages. J. Hazard Mater..

[13] Borgatta M., Breider F. (2024). Inhalation of Microplastics-A toxicological complexity. Toxics.

[14] Stricker A., Hilpmann S., Mansel A., Franke K., Schymura S. (2023). Radiolabeling of Micro-/Nanoplastics via In-Diffusion. Nanomaterials.

[15] Deng Y., Zhang Y., Lemos B., Ren H. (2017). Tissue accumulation of microplastics in mice and biomarker responses suggest widespread health risks of exposure. Sci. Rep..

[16] Nzimande M.C., Mtibe A., Tichapondwa S., John M.J. (2024). A review of weathering studies in plastics and biocomposites-effects on mechanical properties and emissions of volatile organic compounds (VOCs). Polymers.

[17] Du S., Zhu R., Cai Y., Xu N., Yap P.S., Zhang Y., He Y., Zhang Y. (2021). Environmental fate and impacts of microplastics in aquatic ecosystems: a review. RSC Adv..

[18] Jeon S., Lee W.S., Song K.S., Jeong J., Lee S., Kim S., Kim G., Kim J.S., Jeong J., Cho W.S. (2023). Differential particle and ion kinetics of silver nanoparticles in the lungs and biotransformation to insoluble silver sulfide. J. Hazard Mater..

[19] Jeon S., Kim S.H., Jeong J., Lee D.K., Lee S., Kim S., Kim G., Maruthupandy M., Cho W.S. (2021). ABCG1 and ABCG4 as key transporters in the development of pulmonary alveolar proteinosis by nanoparticles. J. Hazard Mater..

[20] Jeon S., Lee D.K., Jeong J., Yang S.I., Kim J.S., Kim J., Cho W.S. (2021). The reactive oxygen species as pathogenic factors of fragmented microplastics to macrophages. Environ. Pollut..

[21] Lee D.K., Jeon S., Jeong J., Song K.S., Cho W.S. (2020). Carbon nanomaterial-derived lung burden analysis using UV-Vis spectrophotometry and proteinase K digestion. Part. Fibre Toxicol..

[22] Jeon S., Kim G., Jeon J.H., Yang S.I., Lee K., Park J.W., Cho W.S. (2026). A simple and reliable method for the qualitative and quantitative analysis of nano- and microplastics in organs for controlled preclinical studies. Part. Fibre Toxicol..

[23] Hamanaka R.B., Mutlu G.M. (2025). Particulate matter air pollution: effects on the respiratory system. J. Clin. Investig..

[24] Leikauf G.D., Kim S.H., Jang A.S. (2020). Mechanisms of ultrafine particle-induced respiratory health effects. Exp. Mol. Med..

[25] Sun X., Chen B., Li Q., Liu N., Xia B., Zhu L., Qu K. (2018). Toxicities of polystyrene nano- and microplastics toward marine bacterium Halomonas alkaliphila. Sci. Total Environ..

[26] Hu M., Palic D. (2020). Micro- and nano-plastics activation of oxidative and inflammatory adverse outcome pathways. Redox Biol..

[27] Kumar P., Nagarajan A., Uchil P.D. (2018). Analysis of cell viability by the lactate dehydrogenase assay. Cold Spring Harb. Protoc..

[28] Kari S., Subramanian K., Altomonte I.A., Murugesan A., Yli-Harja O., Kandhavelu M. (2022). Programmed cell death detection methods: a systematic review and a categorical comparison. Apoptosis.

[29] Hiraiwa K., van Eeden S.F. (2013). Contribution of lung macrophages to the inflammatory responses induced by exposure to air pollutants. Mediat. Inflamm..

[30] Tapak M., Sadeghi S., Ghazanfari T., Mosaffa N. (2023). Chemical exposure and alveolar macrophages responses: 'the role of pulmonary defense mechanism in inhalation injuries**'**. BMJ Open Respir. Res..

[31] Halliwell B., Gutteridge J.M. (2015).

[32] Duan J., Li Y., Gao J., Cao R., Shang E., Zhang W. (2022). ROS-mediated photoaging pathways of nano- and micro-plastic particles under UV irradiation. Water Res..

[33] Gao J., Yang Q., Fan X., Zhou X., Ren P. (2025). Exploring different toxic effects of UV-Aged and bio-aged microplastics on growth and oxidative stress of Escherichia coli. Toxics.

[34] Shafea L., Goebel M.-O., Woche S.K., Peth S. (2025). UV-ageing effects on polystyrene microplastics surface polarity and transport in soils. Environ. Chall..

[35] Thirunavukkarasu G.K., Motlochová M., Bavol D., Vykydalová A., Kupčík J., Navrátil M., Kirakci K., Pližingrová E., Dvoranová D., Šubrt J. (2025). Insights in photocatalytic/fenton-based degradation of microplastics using iron-modified titanium dioxide aerogel powders. Environ. Sci. Nano.

[36] Kadac-Czapska K., Osko J., Knez E., Grembecka M. (2024). Microplastics and oxidative stress-current problems and prospects. Antioxidants.

[37] Lee S.E., Yi Y., Moon S., Yoon H., Park Y.S. (2022). Impact of Micro- and nanoplastics on mitochondria. Metabolites.

[38] Albright J.M., Holian A. (2025). Contribution of particle-induced lysosome membrane permeabilization to NLRP3 inflammasome activation and mitochondrial ROS production. Toxicol. Sci..

[39] Chen J., Chen X., Xuan Y., Shen H., Tang Y., Zhang T., Xu J. (2023). Surface functionalization-dependent inflammatory potential of polystyrene nanoplastics through the activation of MAPK/NF-kappaB signaling pathways in macrophage raw 264.7. Ecotoxicol. Environ. Saf..

[40] Busch M., Bredeck G., Waag F., Rahimi K., Ramachandran H., Bessel T., Barcikowski S., Herrmann A., Rossi A., Schins R.P.F. (2022). Assessing the NLRP3 inflammasome activating potential of a large Panel of Micro- and nanoplastics in THP-1 cells. Biomolecules.

[41] Tenorio M., Graciliano N.G., Moura F.A., Oliveira A.C.M. (2021). Goulart MOF: **N-An-aylcysteine (NAC): impacts on human Health**. Antioxidants.

[42] Mlejnek P. (2022). Direct interaction between N-Acetylcysteine and cytotoxic Electrophile-An overlooked in vitro mechanism of protection. Antioxidants.

[43] Cheng F., Zhang T., Liu Y., Zhang Y., Qu J. (2021). Non-negligible effects of UV irradiation on transformation and environmental risks of microplastics in the water environment. J. Xenobiot.

[44] El Hayek E., Castillo E., In J.G., Garcia M., Cerrato J., Brearley A., Gonzalez-Estrella J., Herbert G., Bleske B., Benavidez A. (2023). Photoaging of polystyrene microspheres causes oxidative alterations to surface physicochemistry and enhances airway epithelial toxicity. Toxicol. Sci..

[45] Kessler A., Hedberg J., Blomberg E., Odnevall I. (2022). Reactive oxygen species formed by metal and metal oxide nanoparticles in physiological Media-A review of reactions of importance to nanotoxicity and proposal for categorization. Nanomaterials.

[46] Canaparo R., Foglietta F., Limongi T., Serpe L. (2020). Biomedical applications of reactive oxygen species generation by metal nanoparticles. Materials.

[47] Ali M.R.K., Wu Y., Tang Y., Xiao H., Chen K., Han T., Fang N., Wu R., El-Sayed M.A. (2017). Targeting cancer cell integrins using gold nanorods in photothermal therapy inhibits migration through affecting cytoskeletal proteins. Proc. Natl. Acad. Sci. U. S. A..

[48] Zhao Y., Wang Y., Ran F., Cui Y., Liu C., Zhao Q., Gao Y., Wang D., Wang S. (2017). A comparison between sphere and rod nanoparticles regarding their in vivo biological behavior and pharmacokinetics. Sci. Rep..

[49] Shahhoseini E., Feltis B.N., Nakayama M., Piva T.J., Pouniotis D., Alghamdi S.S., Geso M. (2019). Combined effects of gold nanoparticles and ionizing radiation on human prostate and lung cancer cell migration. Int. J. Mol. Sci..

[50] Xu X., Xu S., Wan J., Wang D., Pang X., Gao Y., Ni N., Chen D., Sun X. (2023). Disturbing cytoskeleton by engineered nanomaterials for enhanced cancer therapeutics. Bioact. Mater..

[51] Kladko D.V., Falchevskaya A.S., Serov N.S., Prilepskii A.Y. (2021). Nanomaterial shape influence on cell behavior. Int. J. Mol. Sci..

[52] Schinwald A., Chernova T., Donaldson K. (2012). Use of silver nanowires to determine thresholds for fibre length-dependent pulmonary inflammation and inhibition of macrophage migration in vitro. Part. Fibre Toxicol..

[53] Riediker M., Zink D., Kreyling W., Oberdorster G., Elder A., Graham U., Lynch I., Duschl A., Ichihara G., Ichihara S. (2019). Particle toxicology and health - where are we?. Part. Fibre Toxicol..

[54] Lee D.K., Kim G., Maruthupandy M., Lee K., Cho W.S. (2024). Multimodal pulmonary clearance kinetics of carbon black nanoparticles deposited in the lungs of rats: the role of alveolar macrophages. Part. Fibre Toxicol..

[55] Jung W., Yang M.J., Kang M.S., Kim J.B., Yoon K.S., Yu T., Yoon C., Yang H.W., Choi S.J., Park E.J. (2025). Chronic lung tissue deposition of inhaled polyethylene microplastics may lead to fibrotic lesions. Toxicol. Rep..

[56] Yang S., Zhang T., Ge Y., Yin L., Pu Y., Liang G. (2024). Inhalation exposure to polystyrene nanoplastics induces chronic obstructive pulmonary disease-like lung injury in mice through multi-dimensional assessment. Environ. Pollut..

[57] Tomonaga T., Higashi H., Izumi H., Nishida C., Kawai N., Sato K., Morimoto T., Higashi Y., Yatera K., Morimoto Y. (2024). Investigation of pulmonary inflammatory responses following intratracheal instillation of and inhalation exposure to polypropylene microplastics. Part. Fibre Toxicol..

