# Supplementary material for: Environmentally relevant aged nanoplastics amplify oxidative stress–associated inhalation toxicity and delay lung clearance
Source: Redox Biol. 2026 Jun 27;95:104281. doi: 10.1016/j.redox.2026.104281 (PMC13331772; doi:10.1016/j.redox.2026.104281)
Supplement: Multimedia component 1 [file mmc1.docx]

Supporting Information for

**Environmentally relevant aged nanoplastics amplify oxidative stress–associated inhalation toxicity and delay lung clearance**

Soyeon Jeon^1^, Jun Hui Jeon^2^, Gyuri Kim^1^, Sung Ik Yang^2, *^, Wan-Seob Cho^1, *^

^1^Lab of Toxicology, Department of Health Sciences, The Graduate School of Dong-A University, 37, Nakdong-daero 550 beon-gil, Saha-gu Busan, 49315, Republic of Korea

^2^Department of Applied Chemistry, Kyung Hee University, Yongin-si, 17104, Republic of Korea

*Corresponding authors:

Professor Sung Ik Yang ([siyang@khu.ac.kr](mailto:siyang@khu.ac.kr)); Professor Wan-Seob Cho ([wcho@dau.ac.kr](mailto:wcho@dau.ac.kr))


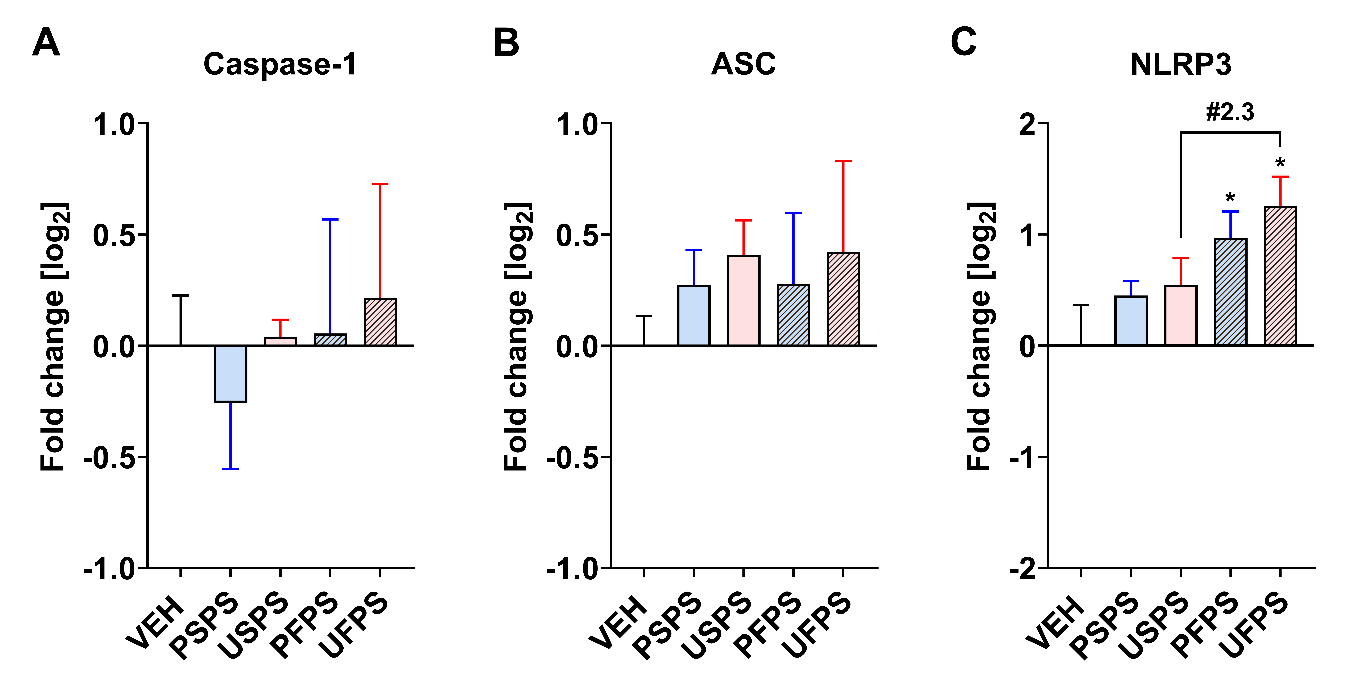
 Figure S1. Gene expression in alveolar macrophages 24 h after pharyngeal aspiration. mRNA expression levels of (A) caspase-1, (B) apoptosis-associated speck-like protein containing a CARD (ASC), and (C) NLR family pyrin domain containing 3 (NLRP3) were analyzed in alveolar macrophages collected from bronchoalveolar lavage fluid (BALF) by real-time PCR. PSPS, pristine spherical polystyrene; USPS, UV-irradiated spherical polystyrene; PFPS, pristine fragmented polystyrene; UFPS, UV-irradiated fragmented polystyrene. Data are presented as mean ± standard deviation (SD) (*n* = 4). Statistical significance was determined by one-way ANOVA followed by Tukey’s post hoc test (^*^*p* < 0.05 vs. VEH; ^#^*p* < 0.05 vs. USPS).


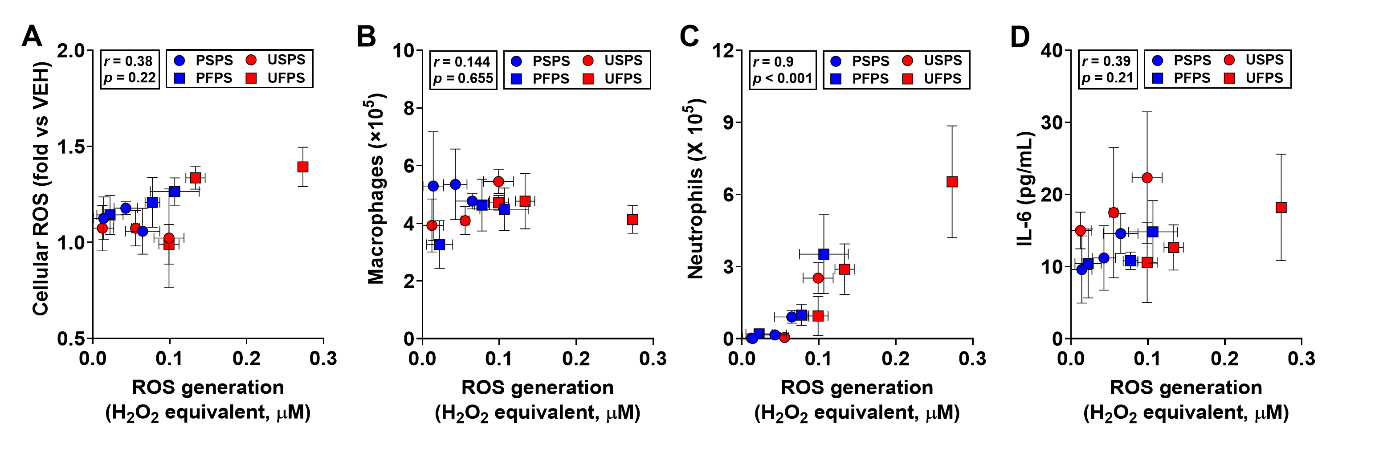
Figure S2. Correlation with the oxidative potential of test nanoplastics and the lung inflammation parameters. The Spearman’s correlation test of the intrinsic reactive oxygen species (ROS) of nanoplastics against (A) cellular ROS, (B) number of macrophages, (C) number of neutrophils, and (D) interleukin (IL)-6. PSPS, pristine spherical polystyrene; USPS, UV-irradiated spherical polystyrene; PFPS, pristine fragmented polystyrene; UFPS, UV-irradiated fragmented polystyrene. Spearman’s correlation analyses between intrinsic ROS-generating capacity of nanoplastics and pulmonary inflammatory parameters in bronchoalveolar lavage fluid (BALF) 1 day after a single pharyngeal aspiration in mice. Data are presented as mean ± SD (*n* = 4).


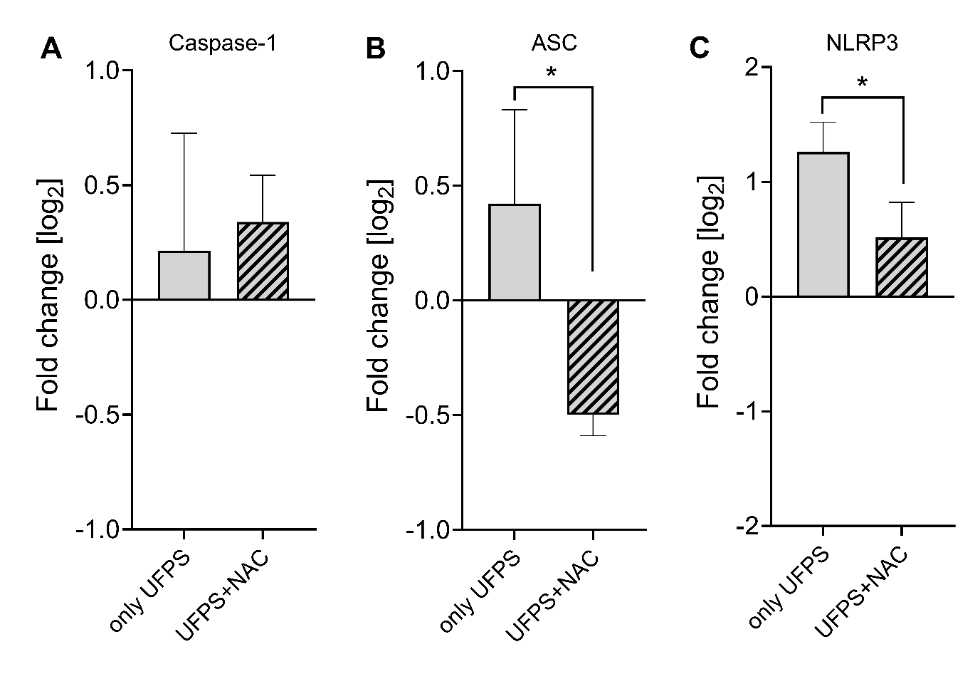


Figure S3. Effects of N-acetyl cysteine (NAC) pretreatment on NLRP3 inflammasome induced by UFPS. UFPS was pretreated with NAC (UFPS vs. NAC-pretreated UFPS), and gene expression of the NLRP3 inflammasome complex was evaluated by bronchoalveolar lavage fluid (BALF) analysis 1 day after a single pharyngeal aspiration in mice at a dose of 100 µg/mouse. Gene expression of (A) caspase-1, (B) ASC, and (C) NLRP3 was measured in alveolar macrophages isolated from BALF. ASC, apoptosis-associated Speck-like protein containing a CARD; NLRP3, NLR family pyrin domain containing 3; UFPS, UV-irradiated fragmented polystyrene. Data are presented as mean ± SD (*n* = 4). ^*^*p* < 0.05 indicates a statistically significant difference between groups, as determined by the Mann-Whitney U test.


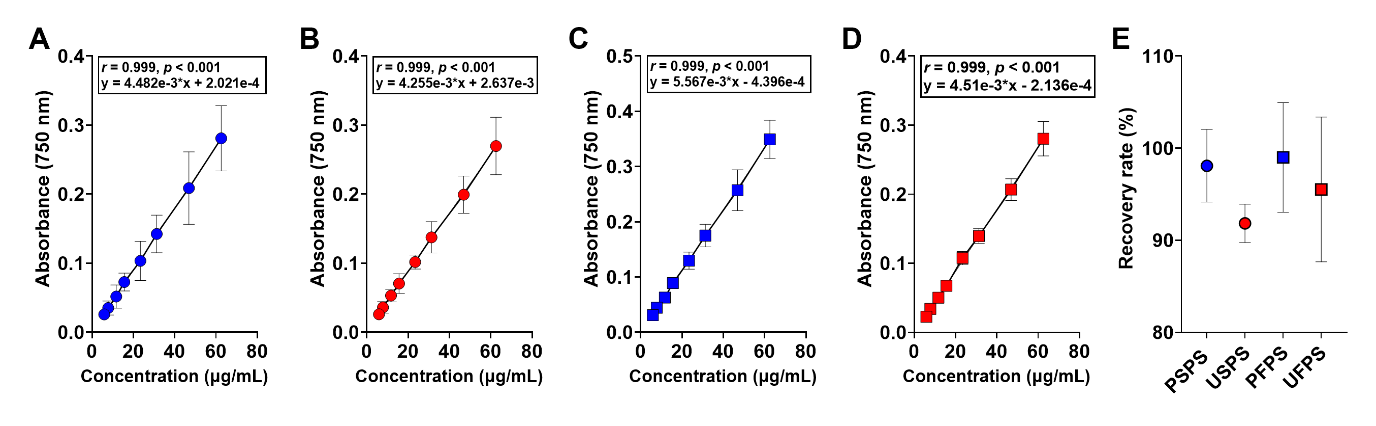


Figure S4. Standard curve fit of all test nanoplastics. The absorbance (750 nm) of (A) pristine spherical polystyrene (PSPS), (B) UV-irradiated spherical polystyrene (USPS), (C) pristine fragmented polystyrene (PFPS), and (D) UV-irradiated fragmented polystyrene (UFPS) was measured in distilled water (DW) using a UV-Vis spectrophotometer. (E) Recovery rate of test nanoplastics. Note that the absorbance-based standard curves of all test nanoplastics exhibited excellent linearity (*r* = 0.999, *p* < 0.001). The recovery rate was more than 92% in all test nanoplastics. Data are presented as mean ± SD (*n* = 4).


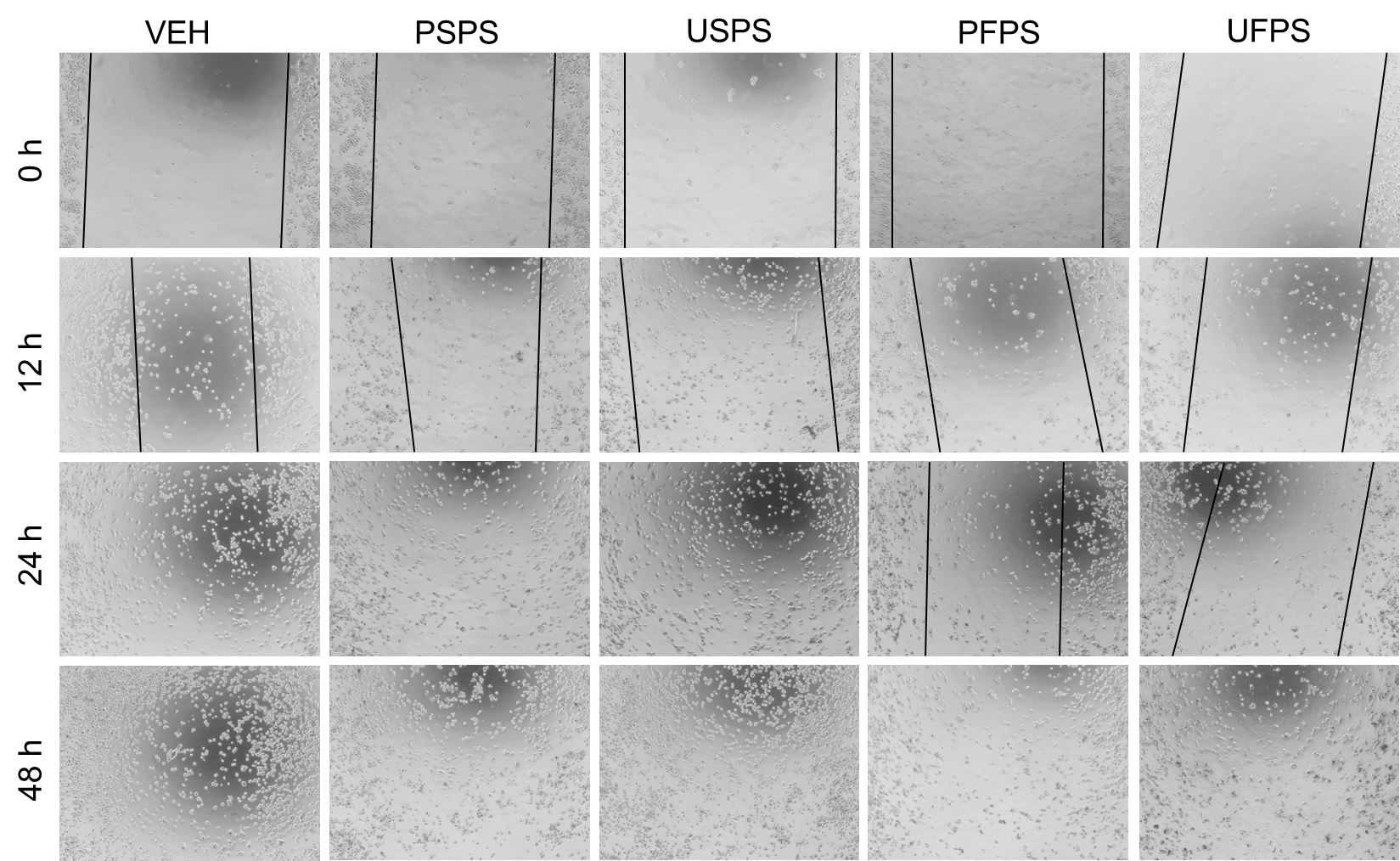


Figure S5. The cell images from the wound-healing assay for evaluating migration across all test nanoplastics. Note that the cell with nanoplastics was incubated for 6 h, then the nanoplastics were washed out, and the cells were observed for 48 h. Spherical-type nanoplastics exhibited markedly faster migration at 24 h, with a wound closure rate comparable to that of the control group. In contrast, cells exposed to fragmented-type nanoplastics migrated more slowly and showed less wound closure. PSPS, pristine spherical polystyrene; USPS, UV-irradiated spherical polystyrene; PFPS, pristine fragmented polystyrene; UFPS, UV-irradiated fragmented polystyrene; VEH, vehicle control.

Table S1. Physicochemical properties of test nanoplastics

| Test  particles | Primary size  (nm) | Hydrodynamic diameter  in PBS (nm) | Zeta potential  in PBS (mV) | Surface area (BET, m^2^/g) | Pore diameter  by BET (nm) |
| --- | --- | --- | --- | --- | --- |
| PSPS | 552.3 ± 97.5 | 579.8 ± 23.9 | -13.3 ± 0.7 | 14.17 | 28.66 |
| USPS | 469.4 ± 47.2 | 547.4 ± 11.2 | -13.5 ± 1.3 | 13.85 | 31.99 |
| PFPS | 462.8 ± 54.2 | 527.3 ± 24.4 | -13.5 ± 0.8 | 15.26 | 25.98 |
| UFPS | 428.7 ± 80.9 | 498.9 ±52.0 | -14.4 ± 0.3 | 11.98 | 26.73 |

BET, Brunauer Emmett Teller; PBS, phosphate-buffered saline; PSPS, pristine spherical polystyrene; USPS, UV-irradiation spherical polystyrene; PFPS, pristine fragmented polystyrene; UFPS, UV-irradiation fragmented polystyrene.

Table S2. XPS Peak fitting and chemical bonding analysis of the test nanoplastics

| Test particles | Peak | Bond | % Area | FWHM (eV) |
| --- | --- | --- | --- | --- |
| PSPS | 284.6 | C-C, C-H | 66.6 | 1.087 |
|  | 284.9 | C=C | 28.4 | 2.165 |
|  | 291.4 | pi-pi* | 4.9 | 1.794 |
|  |  |  |  |  |
| USPS | 284.6 | C-C, C-H | 38.2 | 1.095 |
|  | 285.0 | C=C | 44.3 | 1.922 |
|  | 286.3 | C-O | 9.5 | 2.21 |
|  | 288.0 | C=O | 2.0 | 1.85 |
|  | 291.4 | pi-pi* | 5.9 | 2.492 |
|  |  |  |  |  |
| PFPS | 284.6 | C-C, C-H | 70.4 | 1.137 |
|  | 285 | C=C | 23.3 | 2.743 |
|  | 291.4 | pi-pi* | 6.4 | 2.26 |
|  |  |  |  |  |
| UFPS | 284.5 | C-C, C-H | 59.4 | 1.35 |
|  | 285.0 | C=C | 13.9 | 2.88 |
|  | 286.0 | C-O | 12.6 | 1.82 |
|  | 288.0 | C=O | 10.9 | 3.72 |
|  | 291.4 | pi-pi* | 3.2 | 2.12 |
|  |  |  |  |  |
| UFPS + NAC |  |  |  |  |
|  | 284.53 | C-C, C-H | 54.38 | 1.35 |
|  | 285.32 | C=C | 22.33 | 3.75 |
|  | 285.89 | C-O | 14.69 | 1.62 |
|  | 288.00 | C=O | 7.60 | 1.54 |
|  | 291.20 | pi-pi* | 1.00 | 1.58 |

FWHM, full width at half maximum; NAC, N-acetyl cysteine; PSPS, pristine spherical polystyrene; USPS, UV-irradiation spherical polystyrene; PFPS, pristine fragmented polystyrene; UFPS, UV-irradiation fragmented polystyrene.

Table S3. The detailed differences in neutrophilic inflammatory potency among the test particles in 100 μg/mouse

| Test particles | PSPS | USPS | PFPS | UFPS |
| --- | --- | --- | --- | --- |
| PSPS | *-* | *p* = 0.4  (2.8-fold) | *p* < 0.05  (3.9-fold) | *p* < 0.001  (7.2-fold) |
| USPS | *p* = 0.4  (2.8-fold) | - | *p* = 0.9  (1.4-fold) | *p* < 0.001  (2.6-fold) |
| PFPS | *p* < 0.05  (3.9-fold) | *p* = 0.9  (1.4-fold) | - | *p* < 0.01  (1.9-fold) |
| UFPS | *p* < 0.001  (7.2-fold) | *p* < 0.001  (2.6-fold) | *p* < 0.01  (1.9-fold) | - |

PSPS, pristine spherical polystyrene; USPS, UV-irradiation spherical polystyrene; PFPS, pristine fragmented polystyrene; UFPS, UV-irradiation fragmented polystyrene. *p* < 0.05, *p* < 0.01, and *p* < 0.001 indicate a statistically significant difference between groups, as determined by one-way ANOVA followed by Tukey’s post hoc test.
